# Using focus groups to inform a brief video intervention to reduce public stigma toward Black youth living with psychosis

**DOI:** 10.3389/fpsyt.2023.1210222

**Published:** 2023-09-27

**Authors:** Samantha E. Jankowski, Leah G. Pope, Stephen Smith, Shannon Pagdon, Lisa B. Dixon, Doron Amsalem

**Affiliations:** New York State Psychiatric Institute and Department of Psychiatry, Columbia University Vagelos College of Physicians and Surgeons, New York, NY, United States

**Keywords:** psychosis, stigma, race, focus groups, qualitative

## Abstract

**Objective:**

Black individuals living with psychosis are at risk for stigma and marginalization due to systematic discrimination and barriers to receiving treatment. Social contact-based interventions have the potential to reduce stigma; however, interventions with elements specific to the experiences of Black youth are limited. Therefore, we aimed to gather input from Black youth living with psychosis to develop a social contact-based, brief video intervention to reduce public stigma toward Black youth with psychosis.

**Methods:**

Two 90-min focus groups were conducted with seven young Black individuals ages 18–30 with First Episode Psychosis from OnTrackNY. Participants were asked about their experiences of stigma and racial discrimination, and their perspectives on a video intervention. Focus group transcripts were analyzed using thematic content analysis.

**Results:**

Themes that emerged included: the salience of stigma and racial experiences for some participants and not others; the linking of religiosity and symptoms in Black communities; the importance of taking responsibility for recovery as a coping strategy to counteract stigma; and mixed views on creating a video intervention specific to Black youth.

**Conclusion:**

Meaningful and empowering involvement of individuals with lived experience of psychosis is essential to create stigma reducing interventions. Input from Black youth living with psychosis assisted in developing a culturally tailored brief video-based intervention to reduce public stigma toward Black youth with psychosis that included information about the protagonist’s experience of race and mental illness, specifically family, religious, and community-based experiences.

## Introduction

In the United States, Black individuals living with psychosis are susceptible to systematic discrimination and experience barriers to seeking treatment. For example, rates of psychosis diagnosis are two to four times higher among Black individuals compared to White individuals possibly due to clinician bias and greater vulnerability for psychosis (1–5). Black individuals are also more likely to receive long acting injectable antipsychotic medications, first generation antipsychotics rather than clozapine, and higher doses of medication (6–9). Similarly, they are less likely to receive and utilize essential mental health services (10, 11), even when accounting for income and insurance (12). These disparities put them at risk for stigma, marginalization in employment and social relationships, and unnecessary hospitalizations and medication prescriptions, in the case of misdiagnosis (2, 10).

Stigma is often divided into two types: public- and self-stigma. Public stigma includes negative beliefs that result in fear or discrimination of individuals with mental illness, while self-stigma is the internalization of these negative beliefs by those with mental illness (13, 14). Both types of stigma have the potential to be exacerbated by the interaction of having both a mental illness and marginalized identity (15, 16). To address this overlap, Oexle and Corrigan (15) recommend culturally tailored interventions in order to reduce stigma in vulnerable groups. Social-contact-based interventions, which involve interpersonal contact with individuals of a stigmatized group and balance discussion of struggles and recovery, are most effective at reducing stigma (17, 18). Notably, video-based interventions have similar efficacy to in-person contacts (19, 20).

In our prior research, we conducted randomized control trials that showed the efficacy of utilizing brief social-contact based video interventions to reduce public stigma among young adults toward individuals living with psychosis (21–26). Brief online interventions are easier to distribute, less costly, and can be targeted toward youth due to their high use of online platforms. Targeting studies toward youth is essential due to their overlapping age with their initial experiences of psychosis and possibility of intervening before stigmatizing attitudes are solidified. One of our prior studies, which examined the impact of tailoring the video narrative to gender, found a greater stigma reduction effect for women when placed in a video intervention group where a female presenter discussed gender-related themes as compared to when she did not (26). This finding suggests that tailoring the narrative for specific characteristics can bolster stigma reduction; however, no studies have examined the impact of including racialized experiences. Additionally, stigma reduction studies targeting Black individuals are limited, with only one study comparing video and in person contact of a person with panic disorder for Black undergraduates (27, 28). Therefore, there is a need for culturally tailored video interventions including elements specific to the experiences of Black youth with psychosis.

In order to tailor such interventions appropriately, it is essential to gain a better understanding of the intersection of stigma related to mental illness and experiences of racial discrimination for young Black individuals. Prior qualitative studies have been limited to stigmatizing experiences of Black men ages 19–38 with first episode psychosis (FEP) who entered treatment through coercive pathways (29), experiences of family members (30), or studies conducted outside of the United States (31). To our knowledge, no prior study has collected data to inform the development of a brief social contact based video. Therefore, we conducted focus groups with young Black individuals ages 18–30, living in the US, who were recently diagnosed with FEP in order to have a better understanding of the intersection of stigma and racial experiences. We then describe the process of video script development for a brief social contact video to reduce public stigma toward young Black individuals with psychosis.

## Methods

### Recruitment and data collection

We conducted two 90-min focus groups with seven young Black individuals from OnTrackNY with FEP in May and June 2022. OnTrackNY is a 2 year coordinated specialty care program that provides early intervention treatment targeting school, work, and social relationships to youth ages 16 to 30 who are within 2 years of experiencing a first episode of nonaffective psychosis (32). The program is recovery-focused, utilizing a shared decision making framework (32). Participants were eligible to participate in the focus groups if they were Black, between the ages of 18–30, self-identified as being diagnosed with psychosis, and were currently enrolled in the OnTrackNY program. OnTrack has 800 active participants, of which 34% are Black. Participants were assessed by a clinician to have a diagnosis of psychosis upon enrollment in OnTrack and were referred to the study by OnTrack clinicians and a youth council comprised of OnTrack participants and program graduates. The project was approved by the Institutional Review Board of the New York State Psychiatric Institute. Participants provided informed consent and were compensated $50 for study participation. Focus groups were conducted via HIPAA-compliant Zoom by one of the co-authors, a Black clinical psychologist.

A semi-structured focus group guide was developed to explore experiences of stigma and racial discrimination, and to collect perspectives on the development of a video intervention to reduce public stigma toward Black youth with psychosis. During the focus groups, participants were first asked to describe their experiences of stigma and racial discrimination and the intersectionality of being Black and having psychosis. Then they were asked to provide feedback on a brief video featuring a Black presenter who discussed his experiences living with psychosis. They were asked about their feelings regarding the protagonist, and whether he should include more information about his racial experiences. Between the first and second focus groups, the focus group guide was also updated to include more specific questions about racial identity, describing mental health to others in their community, and asking what elements would be relatable for a Black audience.

### Data analysis

Focus groups were audio-recorded and transcribed. The research team individually coded the transcripts using deductive and inductive approaches and refined these codes during team meetings (33). The research team used data collected from these focus groups to inform the development of a script for a brief video intervention that will be studied in a subsequent randomized control trial to reduce public stigma toward Black individuals with psychosis.

## Results

Participants ranged from 18 to 27 years old with a median age of 22. There were 3 women and 4 men. All participants identified as Black and were enrolled in OnTrackNY. Four main themes emerged: (1) the salience of stigma and racial experiences for some participants and not others, (2) the linking of religiosity and symptoms, (3) the importance of taking responsibility for recovery, and (4) mixed views on creating a video intervention specific to Black youth.

### Stigma and race-related experiences

Participants had varied views regarding the overlap between stigma and race-related experiences. Some participants reported that they did not experience stigma or race-related discrimination related to their mental illness. They reported having positive treatment experiences, including “being around other people who were open about their problems” and feeling empowered to “vocaliz[e] my opinions and saying that I do not like certain things being said.” They attributed lack of stigma to a general societal shift where people are more accepting of mental illness or due to not telling anyone outside of family about their experience. One participant acknowledged “when you are a Black person, there’s more stigma in my opinion,” but stated that they did not directly experience stigma or discrimination.

Those who did experience stigma noted an initial fear of seeking help due to how others may perceive them, including being viewed “as childish or a baby” or being hospitalized “like in movies.” When asked specifically about experiences as a Black person with schizophrenia, participants primarily focused on their experiences with family or in their communities. For example, they reported that family members would insinuate that they were having symptoms intentionally, due to “not being busy enough,” or due to a “punishment from a higher power.” The role of religion is discussed further below. Regarding the community, one participant noted “I’ve heard other Black people say that mental illness is just like a cowardly thing almost or that it’s not real, same things I’ve heard from my family pretty much.” One participant also mentioned unique difficulties as a Black male, due to the need to “carry yourself, not express too much and no emotion” to avoid “being judged or perceived as weak.”

Other participants saw more direct parallels between mental health stigma and racial discrimination. “Black people are already demonized for their demeanor anyway,” reflected one man, noting that Black men are often assumed to be dangerous, unfit parents, or “animalistic,” while Black women are viewed as “loud, unstable, unfeminine.” He connected these views with common stereotypes about individuals with mental illness. Another noted that there are “a lot of historical connections between mental health and racism. The fact that people kind of call individuals ‘insane’ and, you know, these terms are all from like the ability to control and not control someone and like control their actions in my opinion.”

### Religious beliefs and mental health stigma

When participants were asked about difficulties of being a Black person with mental illness and situations where they felt misunderstood, a common theme that emerged was having other individuals within the Black community view the participants’ symptoms as a spiritual, rather than mental health concern. For example, some participants noted that early in their illness, family members believed that their symptoms were due to “a punishment from a higher power” or “not being raised religiously.” Another noted, “it was just a lot of judgement and people wanted you to go to church. And that’s one thing in the Black community, I think, is like pretty deep too. Like people just say, ‘oh like you probably have a demon or something [rather than a mental health concern].’” When asked about specific elements to include in a video geared toward a Black audience, participants reported that it would be important to include these references to religion due to a connection between mental illness and demonic possession that is sometimes espoused by Church leaders in the Black community. Finally, one participant advocated for a balanced view where both perspectives are accounted for: “It could be balanced. It could be, you know, he’s going through something mentally and can use religion in it too.”

### Taking responsibility for your mental health as a coping strategy

When asked about what helped them counteract stigma, participants emphasized the importance of surrounding themselves with positive support people and dismissing stereotypes, “learning how to not really care what other, what stranger’s negative perceptions of me are. I do not really walk around telling everybody my problems…what other people have to say about me should not stop my personal journey because I’m left with me.” Another emphasized the importance of seeking help, particularly as a male, and ignoring stereotypes about masculinity that imply that men should not express emotions and are “weak” for seeking help. He noted that it is ok to be “vulnerable and express feelings.”

### Feedback on a video intervention

In order to better inform the development of a new social-contact based video intervention that would specifically target public stigma toward Black youth with psychosis,participants were shown an existing video intervention during focus groups and asked about whether the Black protagonist should have included information about his experiences related to his race. Participants reported that it was most important for them to be able to connect with the protagonist around shared experiences; they wanted to connect with the narrative and presenter on a “humanistic” level. For example, one noted: “I think that a lot of times when people look at these videos of other people talking about their mental health conditions and how they navigated, they are looking to relate, they are looking to see how other people navigated their mental health issues.” One also noted that including racialized experiences “could’ve been slightly more relatable, only if I had experienced the same things that he did.” Regarding experiences that would be relatable, participants suggested including information discussed above that had been relevant to them as Black youth navigating mental illness—managing family member and community stereotypes as well as confronting the belief that the symptoms of mental illness are related to a lack of religion.

Other participants expressed uncertainty about focusing on the racial experiences of a Black protagonist. Some worried that such a focus could cause non-Black viewers to feel excluded due to the video only having a focus on Black individuals. They thought it would send a message that “his race mostly struggles with [mental illness],” as opposed to mental illness being a universal issue. One also expressed concern about possible discrimination: “I feel like it’s not that Black people is not important. I feel like if you just make it only about us, people can get offended. Because it’s like, well they only think Black people suffer.” The participant felt that if a global perspective with multiple cultures was included in the video, it would serve as a buffer against the video getting “dislikes” and would be relatable to more people.

## Discussion

The current study sought input from Black youth with lived experience of psychosis to develop a culturally tailored brief video-based intervention to reduce public stigma against Black youth living with psychosis. Main themes included: the salience of race-related stigma experiences for some participants and not others, the linking of religiosity and symptoms in Black communities, the importance of taking responsibility for recovery as a coping strategy to counteract stigma, and mixed views about including race-related experiences in a video intervention.

Regarding race-related stigma, some participants reported that such experiences did not dominate their interactions. When asked what it is like to be a Black person living with schizophrenia, participants primarily focused on their experiences with family members and their communities equating symptoms with “not being busy enough” or due to difficulties with spirituality. Other participants equated racial discrimination with stigma, stating that common stereotypes associated with Black individuals as dangerous, unfit parents, and unstable are analogous to those associated with individuals with mental illness. These differences reinforce the notion that Black individuals are a heterogenous group with unique perspectives on how race impacts their lives at the individual and structural level (34). Due to the limited sample size, this finding needs to be further explored in other studies.

Lack of stigma related experiences in some participants is in line with a prior qualitative study conducted with primarily middle-aged Black men, where participants did not endorse stigma experiences and actively encouraged others to seek help (35). The authors attributed their findings to a possible cultural shift where people are more accepting of mental health conditions, which was suggested by participants in our study as well. This is contrary to prior qualitative research conducted with Black individuals with FEP who entered treatment through coercive pathways (29) and others who reported experiencing guilt about their illness (31). Our study participants’ involvement in OnTrackNY, could have served a protective role, since it is a person centered, recovery oriented coordinated specialty care program for individuals with FEP. Findings from the Recovery After an Initial Schizophrenia Episode Early Treatment Program (RAISE-ETP) study found that Black participants in coordinated specialty care (NAVIGATE), a sample from a different recovery oriented program, reported reduced stigma, while participants in community care reported increased stigma (36).

Participants also noted the theme of “taking responsibility” for their mental illness as a coping strategy to reduce stigma, where they reported seeking treatment, surrounded themselves with positive supports and reported “not caring what others think of them.” Interestingly, this theme is similar to that reported by Black participants in a prior qualitative study where they felt responsibility for their condition and reported a sense of failure about not being able to “snap out of it” (31). However, participants in our study placed emphasis on empowerment and hope for the future, rather than guilt. Again, this perspective may have been due to their participation in OnTrackNY, a program which emphasizes recovery and empowerment (32). The theme of responsibility has also been noted as an important part of information campaigns to promote treatment seeking by emphasizing the role of the community to step in (37). It has also been identified in a prior focus group our team conducted with older individuals with serious mental illness (38).

Participants noted that if the video was geared toward a Black audience, it would be essential to include experiences of being misunderstood by other Black community members who equate mental illness with demonic possession or other spiritual issue. This theme has been identified in studies conducted in Black youth with FEP, who reported experiencing guilt about their symptoms due to thinking it was a sin or punishment from God (31) or having community members attribute supernatural forces or black magic as explanations for symptoms (39). Prior research has also suggested that it is also common for Black people to seek help from spiritual leaders prior to seeking medical treatment and to not notify providers if they are concurrently seeing a spiritual leader due to believing that they will equate their beliefs with symptoms (39, 40). A participant in our focus group acknowledged the importance of integrating both avenues and called for a balanced video where both religious and medical/psychological perspectives are considered equally.

Notably, some participants expressed uncertainty about including a Black protagonist who discussed racial experiences in a video intervention due to its potential to make non-Black viewers feel excluded or to foster prejudice against Black people. Others, however, focused on the value of including elements of their identity as Black youth that could be relatable to others—the influence of family members, community perceptions, and religion. This aligns with results from our prior study that found greater stigma reduction among women in a video intervention group where a female presenter discussed how identifying as a woman influenced her experiences as a person living with psychosis as opposed to when she did not mention any gender related experiences and provided generic mental health information (26).

### The video creation process

Building on the information gathered from the focus group and our previous study, our findings on the relevance of gender in video content, and our desire to test the impact of a video tailored to Black youth, we created a brief video for Black youth that included information about the protagonist’s experience of race and mental illness. Themes from the focus groups were used to inform the script for a culturally tailored video intervention targeted toward Black youth. We followed the principle of moderately disconfirming stereotypes, which emphasizes a balanced discussion of struggles and recovery, as this type of intervention has been determined to be more effective than focusing only on symptoms (41). When discussing struggles it has been suggested by researchers to be cautious about including more challenging aspects of mental illness, such as dying by suicide, as this may have unintended effects (20). Therefore, we did not mention content such as potential violence among individuals with mental illness, even though it was brought up in focus groups. We instead focused on briefly describing symptoms “I was hearing voices and seeing things” and changes in functioning (ex. failing grades, not focusing on appearance). Regarding racialized experiences, we included others’ reactions to the participant’s mental illness and skin color, including blame and implying they are lacking the motivation to get better. For example, we included statements such as “you do not get the benefit of doubt from anybody” and “you are acting like this on purpose.” We also included the role of faith-based communities: “People judge you and tell you to go to church. And that’s one thing in the Black community that, I think, is like pretty deep too. Like people just say, oh you probably have a demon or something,” since this was emphasized by participants as essential to include in a video targeted toward a Black audience.

As part of themes that emphasized hope and recovery, research suggests that it is important to “see the person” (i.e., focusing on the individual rather than the diagnosis) and include recovery oriented (i.e., having a good prognosis, living a meaningful life, fostering hope) messages (42). We included “see the person” messages such as “Once I realized that I have an illness but I’m not the illness…things started getting better” and recovery themes such as starting treatment, being more vocal with family when they express an opinion the presenter disagrees with, and being able to be successful and live a normal life. The video’s efficacy in reducing public stigma toward Black youth with psychosis among Black and non-Black youth ages 18–30 will be tested in a separate study (Amsalem et al., under review).

### Limitations

Our study has some limitations. First, our findings are limited to a small sample. Including more participants may have added more depth to our findings and future research should aim to recruit a larger number of individuals from diverse locations. Second, our study was comprised of participants who received treatment in the recovery oriented OnTrackNY program, which limits generalizability of the findings. Participants reported primarily positive treatment experiences and empowerment after being connected to care, which could have been a function of being involved in the program; however it is worth noting that a prior qualitative study with Black males not enrolled in OnTrackNY had similar findings (35). Additionally, it may have been informative to explore whether substance or medication use is associated with stigmatizing experiences or perceptions of psychosis. Third, our findings are limited to individuals who are within 2 years of experiencing FEP. Future studies should examine stigma and discrimination at different phases of coordinated specialty care programs, including after discharge.

## Conclusion

Stigma and racial discrimination are major barriers to care. The input from Black youth living with psychosis assisted in developing a culturally tailored brief video-based intervention to reduce public stigma toward Black youth with psychosis. Meaningful and empowering involvement of individuals with lived experience in the creation of stigma reducing interventions is essential. More studies are needed to examine experiences of stigma and discrimination among young Black individuals and other specific groups. Studies should also examine the efficacy of these videos and how identification with racial identity and perceived discrimination impacts participant responses to culturally tailored interventions.

## Data availability statement

The anonymized data supporting the conclusions of this article will be made available by the authors, without undue reservation.

## Ethics statement

The studies involving humans were approved by New York State Psychiatric Institute. The studies were conducted in accordance with the local legislation and institutional requirements. The participants provided their written informed consent to participate in this study.

## Author contributions

SJ wrote the first draft of this manuscript. SJ, DA, SP, and SS coordinated the study and collected the data. SJ, DA, LP, and SS performed the statistical analyses. SJ, DA, and LP interpreted the results and drafted the manuscript. All authors contributed to the article and approved the submitted version.

## Conflict of interest

The authors declare that the research was conducted in the absence of any commercial or financial relationships that could be construed as a potential conflict of interest.

## Publisher’s note

All claims expressed in this article are solely those of the authors and do not necessarily represent those of their affiliated organizations, or those of the publisher, the editors and the reviewers. Any product that may be evaluated in this article, or claim that may be made by its manufacturer, is not guaranteed or endorsed by the publisher.

## References

[1] Olbert CM Nagendra A Buck B. Meta-analysis of black vs. white racial disparity in schizophrenia diagnosis in the United States: Do structured assessments attenuate racial disparities? J Abnorm Psychol. (2018) 127:104–15. doi: 10.1037/abn0000309, PMID: 29094963

[2] Schwartz RC. Racial disparities in psychotic disorder diagnosis: A review of empirical literature. World J Psychiatry. (2014) 4:133. doi: 10.5498/wjp.v4.i4.133, PMID: 25540728 PMC4274585

[3] Bresnahan M Begg MD Brown A Schaefer C Sohler N Insel B. Race and risk of schizophrenia in a US birth cohort: Another example of health disparity? Int J Epidemiol. (2007) 36:751–8. doi: 10.1093/ije/dym041, PMID: 17440031

[4] Eack SM Bahorik AL Newhill CE Neighbors HW Davis LE. Interviewer perceived honesty as a mediator of racial disparities in the diagnosis of schizophrenia. Psychiatr Serv. (2012) 63:875–80. doi: 10.1176/appi.ps.201100388 22751938 PMC3718294

[5] Anglin DM Ereshefsky S Klaunig MJ Bridgwater MA Niendam TA Ellman LM. From womb to neighborhood: A racial analysis of social determinants of psychosis in the United States. Am J Psychiatry. (2021) 178:599–610. doi: 10.1176/appi.ajp.2020.20071091, PMID: 33934608 PMC8655820

[6] Menand E Moster R. Racial disparities in the treatment of schizophrenia spectrum disorders: How far have we come? Curr Behav Neurosci Rep. (2021) 8:179–86. doi: 10.1007/s40473-021-00236-7

[7] Williams JC Harowitz J Glover J Tek C Srihari V. Systematic review of racial disparities in clozapine prescribing. Schizophr Res. (2020) 224:11–8. doi: 10.1016/j.schres.2020.07.023, PMID: 33183948

[8] Bareis N Olfson M Wall M Stroup TS. Variation in psychotropic medication prescription for adults with schizophrenia in the United States. Psychiatr Serv. (2022) 73:492–500. doi: 10.1176/appi.ps.202000932, PMID: 34587788 PMC8964836

[9] Rost K Hsieh YP Xu S Menachemi N Young AS. Potential disparities in the management of schizophrenia in the United States. Psychiatr Serv. (2011) 62:613–8. doi: 10.1176/ps.62.6.pss6206_0613 21632729

[10] Bommersbach TJ Rhee TG Stefanovics EA Rosenheck RA. Comparison of black and white individuals who report diagnoses of schizophrenia in a national sample of US adults: Discrimination and service use. Schizophr Res. (2023) 253:22–9. doi: 10.1016/j.schres.2021.05.017, PMID: 34088549

[11] Oluwoye O Stiles B Monroe-DeVita M Chwastiak L McClellan JM Dyck D. Racial-ethnic disparities in first-episode psychosis treatment outcomes from the RAISE-ETP study. Psychiatr Serv. (2018) 69:1138–45. doi: 10.1176/appi.ps.201800067, PMID: 30152275 PMC6395511

[12] van der Ven E Susser E Dixon LB Olfson M Gilmer TP. Racial-ethnic differences in service use patterns among young, commercially insured individuals with recent-onset psychosis. Psychiatr Serv. (2020) 71:433–9. doi: 10.1176/appi.ps.201900301, PMID: 31931683

[13] Corrigan PW Rao D. On the self-stigma of mental illness: Stages, disclosure, and strategies for change. Can J Psychiatr. (2012) 57:464–9. doi: 10.1177/070674371205700804, PMID: 22854028 PMC3610943

[14] Parcesepe AM Cabassa LJ. Public stigma of mental illness in the United States: A systematic literature review. Adm Policy Ment Health Ment Health Serv Res. (2013) 40:384–99. doi: 10.1007/s10488-012-0430-z, PMID: 22833051 PMC3835659

[15] Oexle N Corrigan PW. Understanding mental illness stigma toward persons with multiple stigmatized conditions: Implications of intersectionality theory. Psychiatr Serv. (2018) 69:587–9. doi: 10.1176/appi.ps.201700312, PMID: 29385960

[16] Bowleg L. Evolving intersectionality within public health: From analysis to action. Am J Public Health. (2021) 111:88–90. doi: 10.2105/AJPH.2020.306031, PMID: 33326269 PMC7750585

[17] Corrigan PW Michaels PJ Vega E Gause M Larson J Krzyzanowski R. Key ingredients to contact-based stigma change: A cross-validation. Psychiatr Rehabil J. (2014) 37:62–4. doi: 10.1037/prj0000038, PMID: 24417232

[18] Thornicroft G Mehta N Clement S Evans-Lacko S Doherty M Rose D. Evidence for effective interventions to reduce mental-health-related stigma and discrimination. Lancet. (2016) 387:1123–32. doi: 10.1016/S0140-6736(15)00298-6, PMID: 26410341

[19] Corrigan P Michaels PJ Morris S. Do the effects of antistigma programs persist over time? Findings from a meta-analysis. Psychiatr Serv. (2015) 66:543–6. doi: 10.1176/appi.ps.201400291, PMID: 25686817

[20] Janoušková M Tušková E Weissová A Trančík P Pasz J Evans-Lacko S. Can video interventions be used to effectively destigmatize mental illness among young people? A systematic review. Eur Psychiatry. (2017) 41:1–9. doi: 10.1016/j.eurpsy.2016.09.008, PMID: 28049074

[21] Amsalem D Yang LH Jankowski S Lieff SA Markowitz JC Dixon LB. Reducing stigma toward individuals with schizophrenia using a brief video: A randomized controlled trial of young adults. Schizophr Bull. (2021) 47:7–14. doi: 10.1093/schbul/sbaa114, PMID: 33484269 PMC7825082

[22] Amsalem D Markowitz JC Jankowski SE Yang LH Valeri L Lieff SA. Sustained effect of a brief video in reducing public stigma toward individuals with psychosis: A randomized controlled trial of young adults. Am J Psychiatry. (2021) 178:635–42. doi: 10.1176/appi.ajp.2020.20091293, PMID: 33900809

[23] Amsalem D Valeri L Jankowski SE Yang LH Bello I Nossel I. Reducing public stigma toward individuals with psychosis across race and gender: A randomized controlled trial of young adults. Schizophr Res. (2022) 243:195–202. doi: 10.1016/j.schres.2022.03.011, PMID: 35397250

[24] Amsalem D Jankowski SE Pagdon S Valeri L Yang LH Markowitz JC. Selfie videos to reduce stigma and increase treatment seeking among youths: Two noninferiority randomized controlled trials. Psychiatr Serv. (2023) 74:229–36. doi: 10.1176/appi.ps.20220168, PMID: 36254455

[25] Jankowski SE Yanos P Dixon LB Amsalem D. Reducing public stigma towards psychosis: A conceptual framework for understanding the effects of social contact based brief video interventions. Schizophr Bull. (2023) 49:99–107. doi: 10.1093/schbul/sbac143, PMID: 36190348 PMC9810007

[26] Amsalem D Jankowski SE Pagdon S Valeri L Smith S Yang LH. “It is hard to be a woman with schizophrenia”: Randomized controlled trial of a brief video intervention to reduce public stigma in young adults. J Clin Psychiatry. (2022) 84:22m14534. doi: 10.4088/JCP.22m14534, PMID: 36541795

[27] Rivera KJ Zhang JY Mohr DC Wescott AB Bamgbose PA. A narrative review of mental illness stigma reduction interventions among African Americans in the United States. J Ment Health Clin Psychol. (2021) 5:20–31. doi: 10.29245/2578-2959/2021/2.1235, PMID: 34632464 PMC8496896

[28] Vinson ES Abdullah T Brown TL. Mental illness stigma intervention in African Americans: Examining two delivery methods. J Nerv Ment Dis. (2016) 204:400–3. doi: 10.1097/NMD.0000000000000458, PMID: 27078010

[29] Knight S Jarvis GE Ryder AG Lashley M Rousseau C. “It just feels like an invasion”: Black first-episode psychosis patients’ experiences with coercive intervention and its influence on help-seeking Behaviours. J Black Psychol. (2023) 49:200–35. doi: 10.1177/00957984221135377

[30] Franz L Carter T Leiner AS Bergner E Thompson NJ Compton MT. Stigma and treatment delay in first-episode psychosis: A grounded theory study. Early Interv Psychiatry. (2010) 4:47–56. doi: 10.1111/j.1751-7893.2009.00155.x, PMID: 20199480 PMC2860376

[31] Ferrari M Flora N Anderson KK Tuck A Archie S Kidd S. The African, Caribbean and European (ACE) pathways to care study: A qualitative exploration of similarities and differences between African-origin, Caribbean-origin and European-origin groups in pathways to care for psychosis. BMJ Open. (2015) 5:e006562–2. doi: 10.1136/bmjopen-2014-006562, PMID: 25588783 PMC4298103

[32] Bello I Lee R Malinovsky I Watkins L Nossel I Smith T. OnTrackNY: The development of a coordinated specialty care program for individuals experiencing early psychosis. Psychiatr Serv. (2017) 68:318–20. doi: 10.1176/appi.ps.201600512, PMID: 27973999 PMC5846122

[33] Bingham AJ Witkowsky P. Deductive and inductive approaches to qualitative data analysis In: Vanover C Mihas P Saldana J, editors. Analyzing and interpreting qualitative research. Thousand Oaks, California: SAGE Publishing, Inc (2021)

[34] Manning M Byrd D Lucas T Zahodne LB. Complex effects of racism and discrimination on African Americans’ health and well-being: Navigating the status quo. Soc Sci Med. (2023) 316:115421. doi: 10.1016/j.socscimed.2022.115421, PMID: 36270847 PMC12970558

[35] Ward EC Besson DD. African American men’s beliefs about mental illness, perceptions of stigma, and help-seeking barriers. Couns Psychol. (2013) 41:359–91. doi: 10.1177/0011000012447824

[36] Nagendra A Weiss DM Merritt C Cather C Sosoo EE Mueser KT. Clinical and psychosocial outcomes of black Americans in the recovery after an initial schizophrenia episode early treatment program (RAISE-ETP) study. Soc Psychiatry Psychiatr Epidemiol. (2022) 58:77–89. doi: 10.1007/s00127-022-02297-9, PMID: 35932309

[37] Hansen H Stige SH Moltu C Johannessen JO Joa I Dybvig S. “We all have a responsibility”: A narrative discourse analysis of an information campaign targeting help-seeking in first episode psychosis. Int J Ment Health Syst. (2019) 13:32. doi: 10.1186/s13033-019-0289-4, PMID: 31086563 PMC6507175

[38] Amsalem D Rogers RT Stroup TS Dixon L Pope LG. Self-stigma among people with serious mental illnesses: The use of focus groups to inform the development of a brief video intervention. Psychiatr Rehabil J. (2023). doi: 10.1037/prj0000570, PMID: 37227841 PMC10643101

[39] Islam Z Rabiee F Singh SP. Black and minority ethnic groups’ perception and experience of early intervention in psychosis services in the United Kingdom. J Cross-Cult Psychol. (2015) 46:737–53. doi: 10.1177/0022022115575737

[40] Singh SP Brown L Winsper C Gajwani R Islam Z Jasani R. Ethnicity and pathways to care during first episode psychosis: The role of cultural illness attributions. BMC Psychiatry. (2015) 15:287. doi: 10.1186/s12888-015-0665-9, PMID: 26573297 PMC4647639

[41] Reinke RR Corrigan PW Leonhard C Lundin RK Kubiak MA. Examining two aspects of contact on the stigma of mental illness. J Soc Clin Psychol. (2004) 23:377–89. doi: 10.1521/jscp.23.3.377.35457, PMID: 34375138

[42] Clement S Jarrett M Henderson C Thornicroft G. Messages to use in population-level campaigns to reduce mental health-related stigma: Consensus development study. Epidemiol Psichiatr Soc. (2010) 19:72–9. doi: 10.1017/S1121189X00001627, PMID: 20486426

